# Enhanced Selection of High Affinity DNA-Reactive B Cells Following Cyclophosphamide Treatment in Mice

**DOI:** 10.1371/journal.pone.0008418

**Published:** 2010-01-06

**Authors:** Daisuke Kawabata, Jeganathan Venkatesh, Meera Ramanujam, Anne Davidson, Christine M. Grimaldi, Betty Diamond

**Affiliations:** Center for Autoimmune and Musculoskeletal Disease, The Feinstein Institute for Medical Research, Manhasset, New York, United States of America; University of Miami, United States of America

## Abstract

A major goal for the treatment of patients with systemic lupus erythematosus with cytotoxic therapies is the induction of long-term remission. There is, however, a paucity of information concerning the effects of these therapies on the reconstituting B cell repertoire. Since there is recent evidence suggesting that B cell lymphopenia might attenuate negative selection of autoreactive B cells, we elected to investigate the effects of cyclophosphamide on the selection of the re-emerging B cell repertoire in wild type mice and transgenic mice that express the H chain of an anti-DNA antibody. The reconstituting B cell repertoire in wild type mice contained an increased frequency of DNA-reactive B cells; in heavy chain transgenic mice, the reconstituting repertoire was characterized by an increased frequency of mature, high affinity DNA-reactive B cells and the mice expressed increased levels of serum anti-DNA antibodies. This coincided with a significant increase in serum levels of BAFF. Treatment of transgene-expressing mice with a BAFF blocking agent or with DNase to reduce exposure to autoantigen limited the expansion of high affinity DNA-reactive B cells during B cell reconstitution. These studies suggest that during B cell reconstitution, not only is negative selection of high affinity DNA-reactive B cells impaired by increased BAFF, but also that B cells escaping negative selection are positively selected by autoantigen. There are significant implications for therapy.

## Introduction

Systemic lupus erythematosus (SLE) is a systemic autoimmune disease characterized by the production of autoantibodies against a vast array of self antigens, most notably double stranded (ds) DNA [1]. Autoreactive B cells arise routinely in all individuals as a consequence of the molecular processes that govern V gene recombination and B cell receptor (BCR) diversification. In healthy individuals, the B cell repertoire is purged of potentially pathogenic autoreactive B cells at multiple developmental checkpoints; however, in SLE patients, many of these checkpoints are breached and autoreactive B cells become part of the mature, immunocompetent and activated B cell repertoire [2]–[4].

A mainstay of lupus therapy for many decades has been cyclophosphamide (CY), a cytotoxic agent that has been shown to preferentially target B cells [5], [6]. New therapies recently explored for SLE include the use of the anti-CD20 antibody, which selectively depletes B cells [7], [8], as well as autologous hematopoietic stem cell transplantation, which leads to both T and B cell depletion. In each case, the underlying therapeutic strategy is to permit the development of a reconstituted B cell repertoire devoid of autoreactive B cells. It is clear that CY is beneficial in lupus patients. Initial studies of human SLE patients and lupus-prone mouse strains suggested that B cell depletion usually given together with CY ameliorates disease activity in a subset of patients [9], [10], but two large randomized, placebo controlled studies of B cell depletion with anti-CD20 antibody failed to show efficacy at 12 months. There remains a lack of critical information about how autoreactive B cells reconstitute following B cell depletion, especially in light of the observation that serum levels of BAFF rise following B cell depletion [11] in an attempt to restore B cell homeostasis. To begin to address this important issue, we studied the effects of CY-induced B cell depletion on the selection of DNA-reactive B cells in wild type (WT) BALB/c mice and in the R4A Tg BALB/c mouse that expresses the heavy chain of a pathogenic anti-DNA antibody. We demonstrate that during B cell reconstitution, there is an increased maturation of high affinity DNA-reactive B cells resulting in increased serum titers of anti-DNA antibodies. A reduction in the elevated levels of BAFF that result from B cell depletion or a decrease in antigen availability diminished the expansion of these autoreactive B cells.

## Results

### Reconstitution of Splenic B Cell Subsets Following CY Treatment

**Figure 1:**
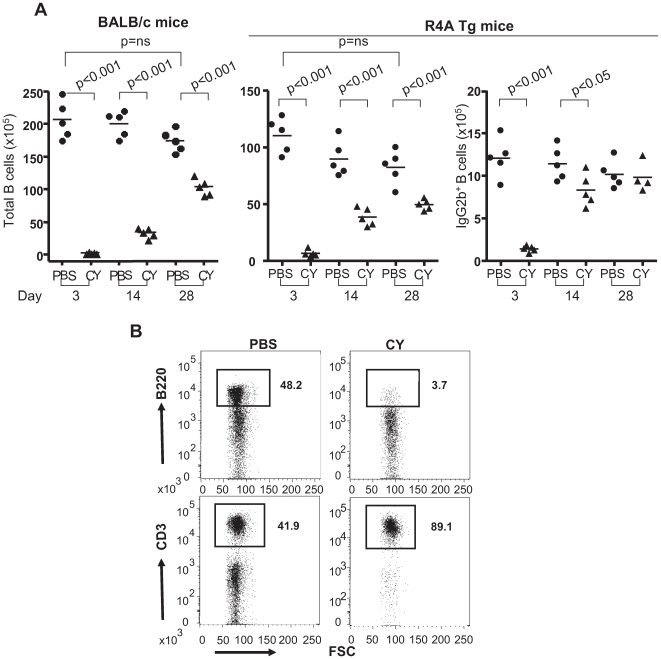
B cells following CY treatment. (A) B cell numbers following CY or PBS treatment. Flow cytometry was performed to identify B (B220^+^) cells and the Tg (IgG2b+) B cells. The B cell numbers from individual mice is represented. A significant decrease in total B cell numbers in both the BALB/c WT mice as well as the BALB/c R4A Tg mice following CY treatment was observed at all time points analyzed, but was near normal by day 28. (B) Lymphocyte numbers following CY or PBS treatment. Flow cytometry was performed to identify B (B220^+^) cells and T (CD3^+^) cells. A representative dot plot is shown.

CY is a DNA alkylating agent that is cytotoxic to hematopoietic cells, most notably B cells [5], [6], and is commonly used to treat patients with lupus nephritis and neuropsychiatric lupus [12]. To establish the kinetics of B cell reconstitution following a single dose of CY (200 mg/kg of body weight), we first examined WT BALB/c mice. As expected, CY-induced B cell depletion was almost complete on day 3 with a greater than 95% reduction in splenic B cells (Figure 1 A&B and Table 1). While CY treatment also depleted T cells, T cell depletion was less extensive than B cell depletion (Figure 1), confirming previous reports that B cells are more susceptible to CY treatment [13].

**Table 1:** Absolute number and percentages of splenic B cells and T cells on day 3.

Treatment	B cells (×10^5^)	B cells (%)	T cells (×10^5^)	T cells (%)
PBS	193 (±34)	49.4 (±3.3)	146.0 (±23.6)	41.0 (±5.4)
CY	8.3* (±2.3)	5.4 (±1.8)	35* (±7.7)	81.4 (±1.9)

*n* = 5 mice per group. Data are presented as the mean±SD.

* p<1×10^−4^.

Having established a dose of CY that induced near total B cell depletion and partial T cell depletion, we determined the pattern of B cell reconstitution. The absolute number of B cells in all subsets increased substantially between days 3 and 14 (Figure 1A and Table 2), there was a greater than 50% repopulation of transitional T1 and T2 subsets, while the number of mature FO and MZ B cells still lagged at this time point, such that total B cell numbers were still reduced by 80% in CY-treated mice compared to PBS-treated mice on day 14.

**Table 2:** Percentage of splenic B cells during reconstitution.

		Transitional B cells		Mature B cells	
		T1	T2	FO	MZ
**Day 3**	**PBS**	24.9 (±3.1)	13.7 (±2.2)	54.8 (±9.2)	5.6 (±0.7)
	**CY**	27.6(±7.3)	15.6(±0.01)	29.6* (±0.05)	18.6* (±4.7)
**Day 14**	**PBS**	19.5 (±2.1)	10.8 (±2.1)	59 (±9.2)	4. 2 (±1.3)
	**CY**	50.1* (±9.8)	29.9* (±6.0)	20.4* (±7.8)	4.5 (±0.01)
**Day 28**	**PBS**	12.6 (±2.7)	10.6 (±1.4)	62.9 (±12.4)	12.9 (±1.7)
	**CY**	12.4(±0.04)	19.8* (±2.8)	63.8 (±8.3)	5* (±1.8)

*n* = 5 mice per group. Data are presented as the mean±SD.

* denotes that the comparison of PBS to CY-treated mice was p<0.001.

**Figure 2:**
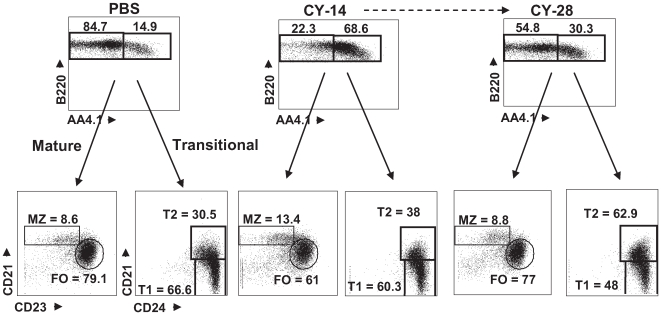
Splenic B cell populations following CY or PBS treatment. The markers B220 and AA4.1 were used to differentiate mature (B220^+^/AA4.1^−^) and transitional (B220^+^/AA4.1^+^) B cell subsets. The mature B cell gate was used to identify marginal zone (CD21^hi^/CD23^−^) and follicular (CD21^hi/^CD23^pos^) populations and the transitional gate was used to identify T1 (CD21^neg^/HSA^hi^) and T2 (CD21^pos^/HSA^hi^) subsets. Five mice were analyzed at each time point. Representative data are shown. The absolute numbers are presented in Table 2.

Between days 14 and 28, total B cell number in CY-treated mice increased to greater than 60% of that in PBS-treated mice; FO and MZ B cell numbers increased to 50% of levels in PBS-treated mice, demonstrating that during this period, restoration of mature splenic B cell compartments was occurring (Table 2 and Figure 2). Taken together, these data indicate that significant reconstitution of mature B cell subsets takes place over a span of approximately 28 days after the existing B cell populations are depleted by a single dose of CY, and are in agreement with previous studies suggesting that B cell numbers return to near normal levels within a month after CY treatment [6].

**Figure 3:**
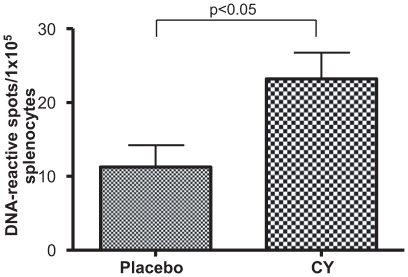
ELISpot assay to enumerate the DNA-reactive B cells. Five BALB/c mice were treated with CY and five with PBS. Student's t test was used to determine the significance between the groups. A significant increase in the DNA-reactive cells was observed in CY-treated mice (p<0.05).

To understand the antigenic specificity of the reconstituting B cell repertoire, we chose to enumerate DNA-reactive B cells. On day 14, we examined the repertoire in WT mice by DNA-specific IgG ELISpot assay. CY-treated mice had a significantly higher frequency of B cells spontaneously secreting anti-DNA antibody than PBS-treated mice (Figure 3). Thus, the reconstituting B cell repertoire was enriched for activated DNA-reactive B cells.

### Expansion of High Affinity DNA Reactive B Cells during Reconstitution in CY-Treated R4A Tg Mice

Since the primary purpose of this study was to determine the effects of CY-induced B cell depletion on the reconstitution of the B cell repertoire of a lupus patient,, we decided to continue our studies using the R4A Tg mouse that expresses the IgG2b heavy chain of the R4A anti-DNA antibody. Most transgene expressing B cells are allelically excluded and express a non-DNA binding antibody or a low affinity DNA binding antibody and display a normal maturational program. There is small number of allelically included (IgM and IgG2b) anergic B cells that express an IgG2b anti-DNA antibody and an IgM antibody that is not DNA-reactive. These cells can only be detected by fusion of LPS-stimulated splenic B cells. We have previously shown that the high affinity DNA-reactive B cells are deleted at both the immature to transitional and transitional to mature stages. Tolerance induction of low affinity DNA-reactive B cells occurs only at the immature to transitional stage. Thus, B cell tolerance is maintained in R4A Tg BALB/c mice and the mice do not express elevated serum titers of anti-DNA antibody despite the enforced expression of the transgenic anti-DNA heavy chain in about 5–10% of B cells. The majority of B cells express an endogenous IgM BCR, allowing for normal competition among B cells for survival niches [14]. On day 3 after CY treatment, R4A Tg-BALB/c mice displayed a greater than 95% loss of splenic B cells, and thereafter mice exhibited a pattern of B cell reconstitution similar to that exhibited by WT BALB/c mice (Table 3 and Figure 1A). The effect of CY treatment on the reconstitution of Tg^+^ (B220^+^/IgG2b^+^) B cells was also determined (Table 4 and Figure 1A). Transgene-expressing B cells of R4A BALB/c mice were reduced on day 3 following CY treatment by greater than 90%. The reconstitution of Tg^+^ B cells compared to Tg^−^ B cells was slightly although not significantly more rapid, resulting in an increased relative frequency of Tg^+^ B cells during reconstitution.

**Table 3:** Percentage of splenic B cells of R4A Tg mice following treatment with CY.

		Transitional B cells		Mature B cells	
		T1	T2	FO	MZ
**Day 3**	**PBS**	8.3 (±2.7)	5.3 (±1.6)	49.7 (±9.2)	25.3 (±9.7)
	**CY**	13.7* (±5.2)	7.7(±3.3)	14.2* (±5.0)	42.5*(±7.8)
**Day 14**	**PBS**	15.2 (±4.3)	8.8 (±2.5)	48 (±6.9)	14.7 (±3.5)
	**CY**	31.5* (±5.9)	21* (±3.8)	27.7* (±7.5)	11.3 (±6.7)
**Day 28**	**PBS**	8.5 (±2.3)	9.5 (±1.0)	47.7 (±14.6)	26.9 (±8.5)
	**CY**	5.0* (±1.0)	9.9 (±4.8)	57.5 (±9.5)	17.3 (±7.6)

*n* = 4–5 mice per group. Data are presented as the mean±SD.

* denotes that the comparison of PBS to CY-treated mice was p<0.001.

**Table 4:** Percentage of IgG2b+ B cells of R4A Tg mice following treatment with CY.

		Transitional B cells		Mature B cells	
		T1	T2	FO	MZ
**Day 3**	**PBS**	13.0 (±1.0)	7.8 (±1.0)	33.0(±2.3)	37.4 (±4.2)
	**CY**	15.4(±3.0)	7.7 (±1.5)	23.1* (±6.9)	46.0 (±7.7)
**Day 14**	**PBS**	16.7 (±6.5)	13.5 (±1.7)	34.1 (±5.6)	30.2 (±4.0)
	**CY**	12.8 (±2.2)	8.4* (±1.0)	37.2 (±4.2)	32.8 (±3.7)
**Day 28**	**PBS**	15.1 (±2.3)	12.3 (±1.8)	37.8 (±7.3)	34.0 (±4.5)
	**CY**	11.2 (±2.7)	13.7 (±2.1)	33.3 (±5.0)	37.3 (±7.2)

*n* = 4–5 mice per group. Data are presented as the mean±SD.

Tg+ B cells were reconstituted in all subsets by day 28. They were significantly reduced in CY-treated mice prior to that time.

* p<0.01.

Our previous analysis of hybridomas from R4A Tg BALB/c have allowed us to identify germline-encoded light chains which, when paired with the R4A heavy chain, give rise to high affinity DNA-reactive B cells. Previously, we have demonstrated that the pairing of the R4A heavy chain with either germline-encoded Vκ1A-Jκ1 or Vκ1A-Jκ4 light chains generates an antibody with high affinity for DNA [15]. Usually, these cells are tolerized, and eliminated during the early stages of selection [16], [17]. To analyze the frequency of high affinity DNA-reactive B cells within the reconstituting repertoire, individual Tg^+^ B cells from 3 individual mice were isolated and single cell RT-PCR and sequence analysis was performed. Tg^+^ B cells were first analyzed to confirm that they expressed a γ2b heavy chain. We also examined Tg^+^ B cells for expression of a μ heavy chain to ensure that we were not seeing a preferential increase in survival of allelically included B cells. Of 15 Tg^+^ B cells isolated by single cell sorting, 14 expressed a γ2b heavy chain; none expressed a μ heavy chain (data not shown). Thus, the cells we studied maintained allelic exclusion. The frequency of mature Tg^+^ B cells utilizing light chains that generate a high affinity anti-DNA antibody was determined. On day 14 the frequency of high affinity DNA-reactive transitional and mature Tg^+^ B cells that were present in CY-treated mice was significantly increased compared to PBS-treated R4A Tg mice (Table 5), indicating that more potentially pathogenic autoreactive B cells bypassed the stages of negative selection in both bone marrow and spleen during reconstitution and entered in the immunocompetent repertoire. Interestingly, on day 28, the frequency of transitional and mature high affinity DNA-reactive B cells was still increased in CY-treated R4A Tg mice; there was a decrease of nearly 50% when compared with day 14, although this difference was not statistically significant (data not shown). These data demonstrate that negative selection was altered in the reconstituting B cells repertoire as there were more high affinity DNA-reactive B cells in the transitional and mature B cell compartments.

**Table 5:** Frequency of high affinity DNA-reactive B cells in CY-treated R4A Tg mice.

	PBS	CY
**Transitional**	4/55 (7.3%)	16/65 (24.4%)*
**Mature**	3/60 (5%)	20/71 (28.2%)*

Data are presented as the frequency of Tg+ B cells expressing Vk1a/Jk1 or Jk4 out of the total number of B cells examined in PBS and CY-treated R4A Tg mice. The percentages are shown in parenthesis. CY-treated mice showed a higher frequency in both the transitional and mature subsets (*p<0.02).

### Elevation in BAFF Following CY Treatment

**Figure 4:**
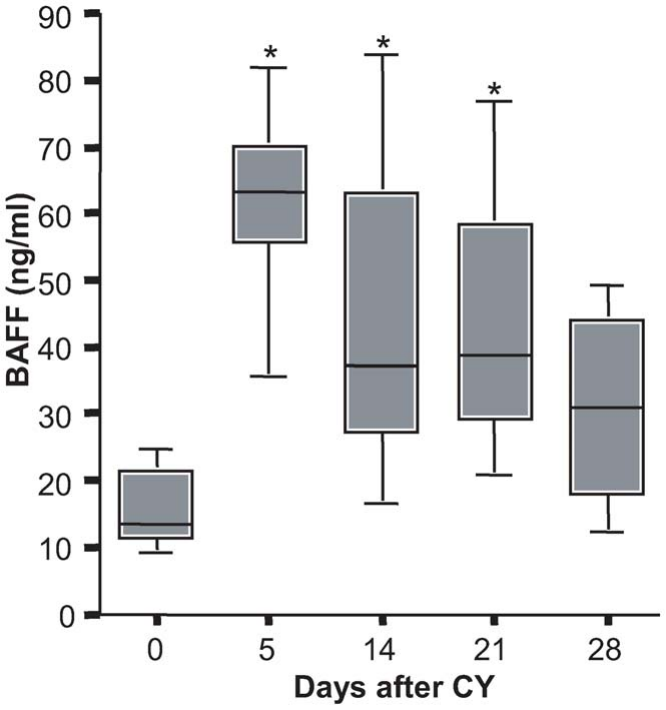
Elevation of serum BAFF during B cell reconstitution. Serum BAFF levels in five mice were measured by ELISA following administration of CY. BAFF was significantly elevated following 3 days after CY treatment and remained elevated until day 28. *p<0.01 as determined by Mann-Whitney test.

BAFF has emerged as a crucial factor that modulates B cell survival and development [18] and is required for the stages of B cell maturation beyond the T1 stage and for the maintenance of FO and MZ B cells [19]–[21]. There are also data to suggest that autoreactive B cells require more BAFF than non-autoreactive B cells for survival and excess BAFF can rescue anergic autoreactive B cells when there is a reduced number of naïve, competitor B cells [22]. It is now established that B cell depletion in humans leads to a rise in serum levels of BAFF [11]. To determine the effects of CY treatment on circulating BAFF levels, serum was obtained from both WT and R4A Tg mice before and after treatment and BAFF levels were quantified by ELISA. In both CY-treated WT BALB/c and R4A Tg BALB/c mice, a significant elevation in BAFF was observed (Figure 4 and data not shown). While the increase in BAFF peaked by day 5, BAFF levels remained significantly increased until day 21 following CY treatment. Thus, these data indicate that serum BAFF levels are markedly elevated during B cell reconstitution.

### BAFF Neutralization Prevented the Accumulation of High Affinity DNA-Reactive B Cells in the Reconstituting Repertoire

The expansion and escape from normal mechanisms of B cell tolerance of high affinity DNA-reactive B cells during B cell reconstitution strongly suggests positive selection of this BCR specificity. To understand the mechanism(s) for the change in B cell selection, we focused on the role of BAFF and autoantigen in the reconstituting repertoire. BAFF is a critical B cell survival factor and BAFF levels have been shown to rise in autoimmune patients following B cell depletion [11], [23]. It has been shown experimentally that increasing the levels of BAFF in a B cell depleted environment can promote the survival of autoreactive B cells that would normally be silenced [22]. We, therefore, assessed the effects of BAFF neutralization on B cell reconstitution and the frequency of high affinity DNA-reactive B cells following CY-induced B cell depletion. R4A Tg mice were given recombinant BAFF-R-Ig as previously described beginning 3 days after CY administration [24]. BAFF-R-Ig treatment resulted in an accumulation of transitional T1 B cells on day 14 (data not shown), as expected, since BAFF is required for maturation to the transitional T2 stage [20]. In addition, neutralization of BAFF resulted in a decrease in MZ B cells, which is also in agreement with previous studies indicating the sensitivity of this B cell subset to changes in BAFF levels [19]. Neutralization of BAFF with BAFF-R-Ig reduced the frequency of immature and mature high affinity DNA-reactive B cells to that present in PBS-treated mice (Table 6), indicating that the emergence of high affinity-DNA reactive B cells into the transitional and then mature B cell repertoire may be due, at least in part, to the increased serum concentration of BAFF in the lymphopenic host.

**Table 6:** Frequency of high affinity DNA-reactive B cells in R4A Tg mice treated with CY and BAFF-R-Ig.

	PBS	CY	CY+BAFF-R-Ig
**Transitional**	5/65 (8%)	16/60 (27%)*	3/60 (5%)
**Mature**	5/64 (8%)	25/70 (36%)*	4/59 (7%)

Data are presented as the frequency of Tg+ B cells expressing Vk1a/Jk1 or Jk4 out of the total number of B cells examined. The percentages are shown in parenthesis. There was an increase in high affinity DNA-reactive B cells in CY vs PBS-treated mice (*p<0.01), but not in CY+BAFF-R-Ig treated mice.

### DNase Treatment Blocks Maturation of High Affinity DNA-Reactive B Cells

DNase treatment of B/W F1 mice has been shown to reduce anti-DNA antibody titers, but there are conflicting data regarding the efficacy of DNase treatment on glomeruloneprhritis and survival [25], [26]. Presumably the reduced production of anti-DNA antibodies reflects a lower amount of DNA to drive the expansion of DNA-reactive B cells.

While it has not been conclusively demonstrated that DNA itself is the eliciting antigen that drives the selection and expansion of DNA-reactive B cells, CY induces massive cell death augmenting the antigenic load potentially capable of stimulating DNA-reactive B cells. We, therefore, asked whether DNase might reduce the expansion of DNA-reactive B cells. We reasoned that DNase-treatment would decrease DNA levels in CY-treated mice, and thus limit the positive selection of high affinity DNA-reactive B cells. Beginning 4 days after R4A mice were given CY, daily injections of active or heat-inactivated DNase were administered for 14 days. Analysis of the DNA-reactive repertoire revealed that the expansion of high affinity DNA-reactive B cells that was observed in CY-treated mice was diminished by administration of active DNase but not by heat-inactivated DNase (Table 7). This was discernable in both the transitional and mature B cell populations, suggesting that the positive selection of autoreactive B cells by antigen begins at the transitional stage of development.

**Table 7:** Frequency of high affinity DNA-reactive B cells in R4A Tg mice treated with CY and DNase.

	PBS	CY	CY+DNase	CY+HI-DNase
**Transitional**	7/65 (10%)	11/52 (21%)*	5/62 (8%)	13/65 (20%)*
**Mature**	4/70 (6%)	14/57 (25%)*	5/50 (10%)	16/70 (23%)*

Data are presented as the frequency of Transgene-positive B cells expressing Vk1a/Jk1 or Jk4 out of the total number of B cells examined. The percentages are shown in parenthesis. There was a significant increase in CY and CY+HI-DNase vs PBS treated mice (*p<0.01), but not in CY+DNase treated mice.

### Anti-DNA Antibody Titers Following CY Treatment

**Figure 5:**
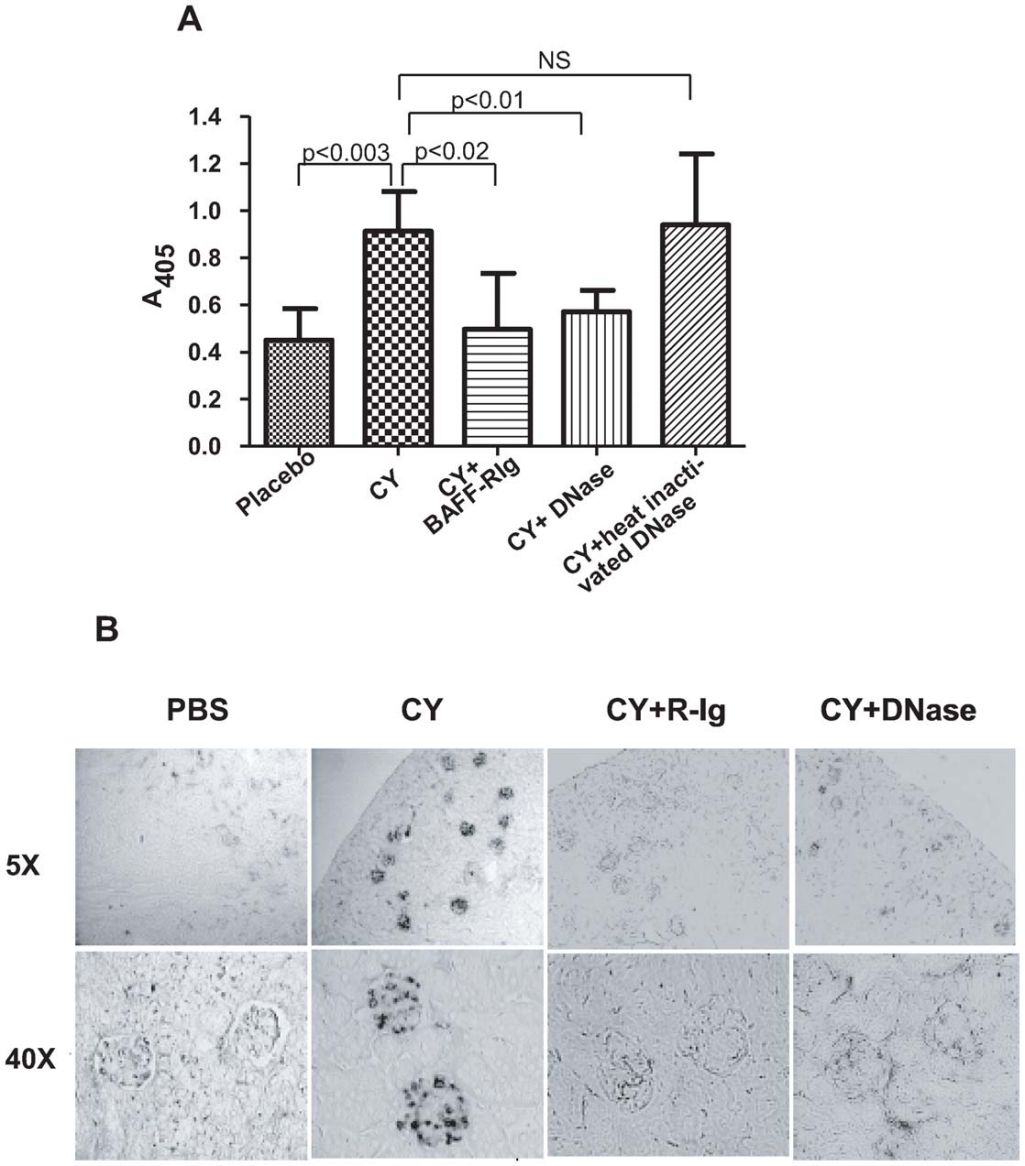
Increase in serum anti-dsDNA antibodies following CY exposure. (**A**) Serum anti-dsDNA antibodies levels in R4A Tg mice following exposure to CY, CY+BAFF-R-Ig, CY+DNase or CY+heat-inactivated DNase (n = 5 mice in each group). A significant increase in dsDNA-reactive antibodies was observed in the serum of CY-treated R4A Tg mice (p<0.003) that was decreased upon treatment wth BAFF-R-Ig (p<0.02) or with active DNase (p<0.01) but not with heat-inactivated DNase. The statistical significance between the groups was determined by paired t test. (**B**) Glomerular Ig deposition in R4A Tg mice following administration of CY, CY+BAFF-R-Ig or CY+DNase. Ig depostion was observed in CY-treated R4A mice and was diminished upon additional treatment with BAFF-R-Ig or with active DNase. Five mice in each group was used for these studies.

In order to determine if the CY-induced skewing of the reconstituting B cell repertoire toward autoreactivity was of consequence, we assayed for anti-DNA antibodies in the serum. CY-treated R4A Tg mice displayed higher titers of anti-DNA antibodies at 28 days than PBS-treated R4A Tg mice (Figure 5A). Furthermore, both BAFF-R-Ig and active DNase, but not heat-killed DNase, led to reduced serum titers of anti-DNA antibody (Figure 5A & 5B). Moreover, glomerular Ig depostion was present in CY-treated mice but not observed in CY-treated mice administered BAFF-R-Ig or active DNase (Figure 5B). Thus, serum DNA reactivity and glomerular Ig deposition confirmed the findings of the repertoire analysis.

## Discussion

The data reported here demonstrate that the eradication of a B cell repertoire may result in the selective expansion of autoreactive B cells and increased autoantibody production. In the WT host, there was a specific expansion of DNA-reactive B cells during B cell reconstitution. We believe this phenomenon might contribute to clinical relapse in patients with SLE. Indeed, these data support a recent clinical study which demonstrated that a subset of lupus patients treated with CY developed anti-phospholipid antibodies and anti-phospholipid syndrome following therapy [27]. They also support a study which analyzed kappa and lambda light chain usage during B cell reconstitution after treatment with rituximab, a B cell depleting antibody, and demonstrated a shift in B cell repertoire toward an increased kappa/lambda ratio [23]. It is noteworthy that in WT non-autoimmune BALB/c mice, the B cell reconstitution phase led to a mature B cell repertoire with a higher frequency of potentially pathogenic DNA-reactive B cells, but no frank serologic autoreactivity. In contrast, in the R4A Tg mouse, predisposed to have enhanced survival of autoreactive B cells, B cell reconstitution was accompanied by autoimmune features including elevated serum titers of autoantibodies and glomerular immunoglobulin deposition. We believe this mirrors the situation in patients with SLE, who are predisposed to a decreased stringency of negative selection, perhaps by virtue of expression of the susceptibility allele of PTPN22 and/or Blk [28]. It is also important to note that elevated BAFF has previously shown to decrease the negative selection of DNA-reactive B cells in mice, although in that study the enhanced BAFF levels were not induced by lymphopenia, and there was no increase in autoantibody titers [29]. We believe this may reflect a difference in fine specificity or affinity of the transgene-encoded anti-DNA antibody such that there was less positive selection.

It is possible that the rise in autoantibody titers that we observe following B cell reconstitution in R4A Tg mice may be transient and at a later time point, the high affinity DNA-reactive B cell population might contract in size as the proportion of non-autoreactive competitor B cells increases. While it is plausible that these potentially pathogenic B cells have a limited time frame in which they can undergo activation, our data indicate that mature high affinity DNA-reactive B cells that are expanded during B cell reconstitution remain part of the naïve repertoire for at least 1 month and can be activated to secrete autoantibody. We believe that once the titers of DNA-reactive antibodies rise, the immune complexes they form activate toll-like receptor 9 (TLR 9) in dendritic cells to increase production of BAFF and proinflammatory cytokines and to transform the dendritic cells from a tolerogenic to an immunogenic state. The same DNA containing immune complexes may activate TLR 9 in DNA-reactive B cells to promote their survival through tolerance checkpoints and to help them class switch to production of the proinflammatory IgG isotype.

The reduction in frequency of high affinity DNA-reactive B cells by treatment with DNase supports a role for antigen in the expansion of these cells after CY exposure. CY treatment increases the concentration of extracellular DNA levels due to the large amount of cell death, so it seems logical to surmise that DNAse may reduce the local concentration of DNA in the spleen and thus, impair the autoantigen-mediated expansion of high affinity DNA-reactive B cells.

In light of the findings that BAFF is elevated in SLE and greatly elevated following B cell depletion and the recent failure of anti-CD20 antibody, a B cell depleting antibody to demonstrate clinical efficacy in the treatment of SLE despite successful B cell depletion, our data suggest that B cell depletion may only be effective if the re-emergence of high affinity autoreactive B cells can be kept in check. In fact, we are concerned that all B cell depleting therapies, whether or not there is an accompanying T cell depletion, may promote the expansion of high affinity autoreactive B cells during B cell reconstitution. This is in keeping with several studies suggesting that high affinity autoreactive B cells have a survival advantage in the absence of a diverse repertoire of competitor B cells [30], [31]. Thus, agents that transiently reduce B cell number may ultimately enhance the survival of potentially pathogenic B cells. Furthermore, the increased BAFF may trigger heavy chain class-switching independent of T cell help and so enhance the pathogenicity of autoantibodies [32]. It is of interest to note that BAFF blockade with an anti-BAFF antibody has just been reported to reduce anti-DNA antibody levels and disease activity in a Phase III study in lupus patients. This therapy causes a mild reduction in B cells, but may limit the survival and maturation of newly minted autoreactive B cells. It will be interesting to determine if it indeed leads to a less autoreactive B cell repertoire.

We are aware that the expansion of high affinity DNA-reactive B cells after CY treatment may not be a consequence of BAFF levels and antigen availability only. There are studies suggesting that the re-emergence of Tregs may be delayed following CY treatment [33]. While our data do not exclude the possibility that a diminished Treg compartment may contribute to the increased maturation of autoreactive B cells, a role for Tregs during selection of the naïve repertoire has not been clearly established.

In conclusion, we demonstrate that expansion of autoreactive B cells occurred following B cell depletion, and BAFF and autoantigen both may play an important role in the enhanced selection of high affinity autoreactive B cells during B cell reconstitution. Our findings raise some critical issues about the therapeutic use of B cell depleting agents and provide a rationale for the use of BAFF blockade during B cell reconstitution to diminish the survival of potentially pathogenic B cells.

## Materials and Methods

### Ethics Statement

Mice were housed in an Association for Assessment and Accreditation of Laboratory Animal Care (AAALAC) approved facility and all experiments were performed under Institutional Animal Care and Use Committee (IACUC) approved protocols.

### Mice and Therapeutic Regimens

BALB/c mice were purchased from Jackson Laboratory (Bar Harbor, ME). R4A BALB/c mice have been described previously [14]. Eight to 12 week-old mice were used for all studies. Mice were housed in a specific pathogen-free facility and animal studies were approved by the Institutional Animal Care and Use Committee at the Feinstein Institute for Medical Research. CY (Cytoxan, Bristol-Meyers Squibb) was dissolved in sterile pyrogen-free PBS and 200 mg/kg body weight was given i.p. Control mice received PBS. Recombinant murine BAFF-receptor-Fc fusion protein (BAFF-R-Ig) was generated as previously described [23]. Beginning 3 days after administration of CY, R4A Tg mice were given 300 µg of BAFF-R-Ig twice a week for 2 or 5 weeks. DNase (450 µg in 200 µl saline) or heat-inactivated DNase (68°C for 15 minutes) (Sigma) was given ip every day for 2 or 5 weeks beginning on day 4 following CY treatment.

### Flow Cytometry

Splenocytes isolated from PBS- and CY-treated mice were stained with fluorochrome-labeled antibodies specific for CD21/CD35, CD23, CD3, CD4, CD8, B220, IgG2b (BD Pharmingen), AA4.1 (eBioscience) and biotinylated mouse IgG2b (Southern Biotech) at 4°C for 30 minutes. Biotinylated mouse IgG2b was detected using streptavidin-conjugated fluorochrome (BD Pharmingen). The cells were then washed with PBS and analyzed by flow cytometry using an LSRII instrument (BD Biosciences) and the data were analyzed using Flowjo software (Tree star).

### Single Cell RT-PCR and Repertoire Analysis

Splenocytes from PBS- or CY-treated R4A mice (3 per condition) were stained with antibodies specific for B220, IgG2b and AA4.1 and B220^+^/Tg^+^/AA4.1^−^ cells were individually sorted into 96-well plates using a FACSAria (BD Biosciences). Single-cell RT-PCR was performed as described previously [4], using the following primers: universal Vκ: 5′GGCTGCAGSTTCAGTGGCAGTGGRTCWGGRAC3′+constant region primer (Cκ) (1^st^ round): 5′TGGATGGGTGGGAAGATG3′ and Cκ (2^nd^ round); 5′AAGATGGATACAGTTGGT3′. PCR products were subjected to exo-SAP treatment (USB Biochemicals) and automated sequencing was performed using the 2^nd^ round Cκ primer (Genewiz Inc., NJ). To confirm heavy chain allelic exclusion in transgene-expressing B cells, PCR of μ constant region was also performed with the following primers: R4A VH primer CTGCAACCGGTGAGGTGAAGCTGGTGGA ATCTG and the μ constant region primers CAGGGGGCTCTCGCAGGAGACGAGG (1^st^ round) and GGGATCCTGGGAAGGACT GACTCTC (2^nd^ round).

### Measurement of Serum BAFF

The concentration of soluble BAFF was determined by ELISA. 96-well plates were coated with 5 µg/ml of anti-mouse BAFF mAb (clone 5A8; Apotech). After blocking with 5% BSA, serial dilutions of mouse serum or mouse recombinant BAFF (Apotech) were added to the wells, followed by 10 µg/ml of biotinylated monoclonal anti-mouse BAFF antibody (clone 1C9; Apotech) and HRP-labeled streptavidin. Optical density was measured at 450 nm.

### Anti-dsDNA ELISA

Sera from PBS- or CY-treated BALB/C mice and from R4A mice treated with PBS, CY, CY+BAFF-R-Ig, CY+DNase or CY+heat inactivated DNase (n = 5 in each group) were diluted 1∶100 and assayed for IgG2b anti-dsDNA antibodies as previously described [2].

### ELISpot Assay

Five BALB/c mice were treated with CY and five with PBS. Splenocytes isolated from these mice were added in serial dilution to DNA-coated plates and incubated for 12 hours at 37°C. Biotin-conjugated goat anti-mouse IgG (Southern Biotechnology) diluted 1∶600 was added, followed by alkaline phosphatase-conjugated streptavidin (Southern Biotechnology) at 1∶1000 dilution. The plates were developed with 5-bromo-4-chloro-3 indolyl phosphate substrate (Sigma-Aldrich). DNA-reactive spots were counted under a dissecting microscope.

### Renal Pathology

Kidneys from R4A Tg mice treated with PBS, CY, CY+BAFF-R-Ig and CY+DNase were fixed in formalin. The fixed tissues were paraffin-embedded, sectioned (10 micron thickness) and stained with biotinylated anti-mouse IgG and developed with alkaline phosphatase ABC detection kit (Vector Laboratories). Glomerular IgG deposition in the kidney sections was visualized under a Zeiss microscope using the Axiovision software. Five mice in each group were used for these studies.

### Statistical Analysis

Statistical analysis was performed using Fishers's exact test, Student's t test and Mann-Whitney Test as indicated in the text. A *p* value of <0.05 was considered to be statistically significant.
